# Evaluation of motor fluctuations in Parkinson’s disease: electronic vs. conventional paper diaries

**DOI:** 10.3389/fneur.2024.1476708

**Published:** 2024-11-22

**Authors:** Kanako Asai, Sayo Kawahara, Emi Shirahata, Kimihiro Iwasaki, Hikoichiro Nakai, Yuta Kajiyama, Seira Taniguchi, Lindun Ge, Keita Kakuda, Yasuyoshi Kimura, Tadashi Miyahara, Hiroki R. Ueda, Kensuke Ikenaka, Hideki Mochizuki

**Affiliations:** ^1^Department of Neurology, Osaka University Graduate School of Medicine, Suita, Japan; ^2^ACCELStars, Bunkyo-ku, Tokyo, Japan; ^3^Department of Neurology, Kawasaki Medical School, Kurashiki, Okayama, Japan; ^4^Department of Systems Pharmacology, Graduate School of Medicine, The University of Tokyo, Bunkyo-ku, Tokyo, Japan; ^5^Laboratory for Synthetic Biology, RIKEN Center for Biosystems Dynamics Research (BDR), Suita, Osaka, Japan; ^6^Department of Systems Biology, Institute of Life Science, Kurume University, Kurume, Fukuoka, Japan

**Keywords:** Parkinson’s disease, motor symptom diary, motor fluctuations, electric device, patient reported outcome measures

## Abstract

**Background:**

Paper symptom diaries are a common tool for assessing motor fluctuations in Parkinson’s disease (PD) patients, but there are concerns about inaccuracies in the assessment of motor fluctuation due to recall bias and poor compliance. We, therefore, developed an electronic diary with reminder and real-time recording functions.

**Objectives and methods:**

To evaluate the effectiveness of the electronic diary, we compared compliance and motor fluctuation assessment with a paper diary. Nineteen PD patients were recruited and recorded paper diaries every 30 min from 8 am to 8 pm for 7 days, followed by 7 days of electronic diary recording using a smartphone and smartwatch. Prior to the recording period, the Parkinson’s Disease Questionnaire (PDQ)-39 and the Movement Disorders Society-sponsored Unified Parkinson’s Disease Rating Scale-Revised (MDS-UPDRS) 1, 2, 3, 4 were measured. Patients completed a patient questionnaire on the usability of the diaries after the recording period.

**Results:**

Total reported time was significantly longer in paper diaries, but there was no significant difference in the number of entries (paper 115 [71–147] vs. electronic 109 [93–116], *p* = 0.77). There was a significant correlation between paper and electronic diaries with respect to motor status. ON time rate recorded in the electronic diary was significantly correlated with PDQ-39, MDS-UPDRS 1, 2, and 4, while MDS-UPDRS 1 was only correlated with ON time rate in the paper diary. The usability of our electronic diary was found to be satisfactory based on the results of patient questionnaire.

**Conclusion:**

Electronic diaries are useful tools that more accurately reflect PD motor fluctuations.

## Introduction

As Parkinson’s disease (PD) progresses, fluctuations in motor and non-motor symptoms can significantly affect quality of life (1). Therefore, PD symptom diaries are widely used in clinical research and medication reconciliation as an important tool to monitor patients’ symptom fluctuations. Prior studies have examined the reliability of paper symptom diaries and have demonstrated the reliability of the patient- or caregiver-reported symptom outcome (2–4). However, current symptom diaries have also raised issues such as low record rates and inaccuracy due to recall bias (5, 6). Therefore, an electronic symptom diary has been developed to record symptoms more accurately and in a real-time manner (7, 8).

A previous study compared the motor status of patients recorded in paper and electronic symptom diaries. It showed no significant differences in ON–OFF status or number of entries between electronic and paper diaries, indicating no advantage of electronic symptom diaries over paper diaries (9).

We focused on recall bias and developed an electronic symptom diary that allows only real-time recording. The purpose of this study is to compare the symptom variability in PD patients recorded by our electronic symptom diaries and by traditional paper symptom diaries and to evaluate the effectiveness of our electronic diary that allowed only real-time recording.

## Materials and methods

### Study protocol approvals and patient consent

The protocol conformed to Helsinki Declaration principles and was approved by the Osaka University review board (approval number: 22311). All participants received written informed consent.

### Participants

This observational study was conducted from May to November 2023. Participants were recruited from PD patients attending the outpatient department of neurology at Osaka University, Japan. The inclusion criteria were as follows: a diagnosis of clinically established or probable PD on the Movement Disorder Society Clinical Diagnostic Criteria for Parkinson’s disease, age 20 years or older, ability to understand and consent to the study, and a history of smartphone use. Exclusion criteria were Mini Mental State Examination (MMSE) score of 26 or less and the inability to use a smartphone.

### Procedures

**Figure 1:**
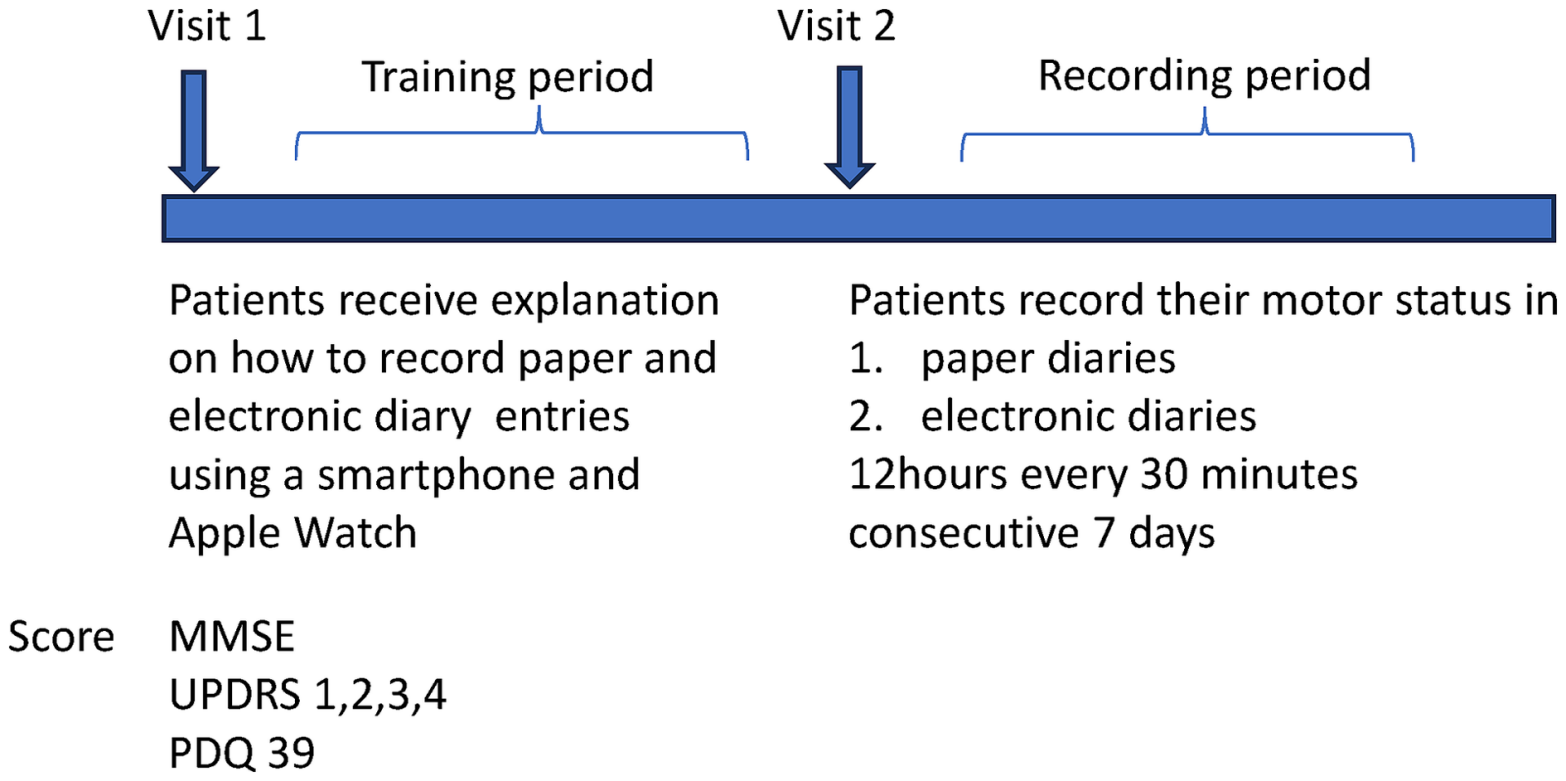
The study protocol. Participants conducted 1 week of recording in each diary after the one-month training period.

The procedures are summarized in Figure 1.

**Visit 1:** Patients were introduced to the electronic symptom diary, with a demonstration to ensure they could operate it effectively. The paper symptom diary was also explained, and patients were asked if they felt confident in filling it as instructed. Movement Disorder Society-Sponsored Revision of the Unified Parkinson’s Disease Rating Scale (MDS-UPDRS) part 1, 2, 3, 4, and The Parkinson’s Disease Questionnaire (PDQ)-39 were investigated.

**Training period:** To allow adequate time for participants, those who already use smartphones, to become familiar with this electronic diary, patients were asked to use both the electronic and paper symptom diaries at home for 1 month for both diaries. There were no restrictions on the frequency of assessments, and patients freely entered their symptoms into either the paper or digital diaries. During this time, they received phone support as needed for any questions or technical issues. This one-month period was deemed sufficient to become familiar with this application.

**Visit 2:** During the training period, we verified that the equipment was used correctly and moved to the recording period.

**Recording period (i)** Paper diary record: A paper symptom diary record was conducted for 7 consecutive days. Patients recorded symptoms every 30 min for 12 h from 8:00 am to 8:00 pm. Patients were allowed to look back and describe their symptoms in accordance with the conventional paper symptom diary recording method.

**Recording period (ii)** Electronic diary record: The patient was subsequently recorded in an electronic symptom diary for seven consecutive days after 7 days paper diary record. Recording was done every 30 min for 12 h from 8:00 a.m. to 8:00 p.m. Every 30 min, an alert with vibration was displayed to prompt recording. Patients were only allowed to record in real-time and were not allowed to look back on past symptoms (Supplementary Figure 1).

Patients completed a patient questionnaire on the usability of the diaries after the recording period (7). The questions in the questionnaire were as follows.

Responses given to the usability questionnaire (%). **Q1**: “The paper diary interfered with my normal activities.” Responses: 1 – Strongly Agree, 2 – Agree, 3 – Sometimes Agree, 4 – Occasionally Agree, 5 – Strongly Disagree. **Q2**: “Using the paper diary system on a daily basis was easy.” Responses: 1 – Strongly Disagree, 2 – Occasionally Agree, 3 – Sometimes Agree, 4 – Agree, 5 – Strongly Agree. **Q3**: “If your doctor wants to use paper diary to monitor your symptoms and adjust your medications, how long would you be willing to record your symptoms?” Responses: 1 – A few days, 2 – 1 week, 3 – 2–3 weeks, 4 – More than 1 month. **Q4**: “The electronic diary interfered with my normal activities.” Responses: 1 – Strongly Agree, 2 – Agree, 3 – Sometimes Agree, 4 – Occasionally Agree, 5 – Strongly Disagree. **Q5**: “Using the electronic diary system on a daily basis was easy.” Responses: 1 – Strongly Disagree, 2 – Occasionally Agree, 3 – Sometimes Agree, 4 – Agree, 5 – Strongly Agree. **Q6**: “If your doctor wants to use electronic diary to monitor your symptoms and adjust your medications, how long would you be willing to record your symptoms?” Responses: 1 – A few days, 2 – 1 week, 3 – 2–3 weeks, 4 – More than 1 month. **Q7**: “I felt comfortable wearing the smartwatch.” Responses: 1 – Strongly Disagree, 2 – Occasionally Agree, 3 – Sometimes Agree, 4 – Agree, 5 – Strongly Agree. **Q8**: “The smartwatch was easy to put on/take off.” Responses: 1 – Strongly Disagree, 2 – Occasionally Agree, 3 – Sometimes Agree, 4 – Agree, 5 – Strongly Agree. **Q9**: “I felt embarrassed wearing the smartwatch.” Responses: 1 – Strongly Agree, 2 – Agree, 3 – Sometimes Agree, 4 – Occasionally Agree, 5 – Strongly Disagree. **Q10**: I experienced technical problems with the electronic diary. Responses: 1 – Strongly Agree, 2 – Agree, 3 – Sometimes Agree, 4 – Occasionally Agree, 5 – Strongly Disagree. Color code from green (score = 1 for least favorable response) to orange (score = 5 for most favorable response).

### About the symptom diary

The paper symptom diary used was Parkinson’s Disease Home Diary (10). Participants were then asked every half-hour time period to indicate their predominant symptom status using the categories of On without dyskinesia, On with non-troublesome dyskinesia, On with troublesome dyskinesia, and Off for seven consecutive days.

In both paper and electronic diary, On with non-troublesome dyskinesia and On without dyskinesia were defined as ON-status. Off was defined as OFF-status. Troublesome dyskinesia was defined as Troublesome dyskinesia-status. Number of entries was defined as the number of times the patient actually recorded the symptom diary. For example, in the paper symptom diary, if the patient described his/her motor status for the last 2 h at once, the number of entries was counted as 1 and the recording time was counted as 2 h. The electronic symptom diary did not permit retrospective entries, so the recording time was 30 min per entry count (Supplementary Figure 2).

We defined “Missing time” as no recordings within 30 min and “Duplicate time” as multiple motor status recordings within 30 min. Both “Missing time” and “Duplicate time” were treated as missing data. “Reporting time” is the number of hours minus “Missing time” and “Duplicate time.” The proportion of motor status was calculated as the percentage of time recorded as troublesome dyskinesia status/ON status/OFF status out of the total input time, excluding missing data (Missing + Duplicate time).

### Outcomes and statistics

The primary outcome was the number of entries in the electronic diary compared to the paper symptom diary. The secondary outcome was the potential association/s between motor fluctuation recorded in each diary and patient-reported outcomes (MDS-UPDRS 1,2,3,4 and PDQ-39). While not all the measures are matched in their recorded time period, this correlational analyses can explore whether they are related measures. Moreover, we evaluated whether the diary type (either paper diary or electronic diary) and number of days were associated with changes in the number of entries recorded. Additionally, we surveyed the patients’ usability questionnaire.

All data are presented as median and interquartile ranges (IQR) or counts and percentages.

Values were compared using the Mann–Whitney U-test for continuous variables and the chi-square test for categorical variables. Spearman correlation coefficient was used to correlate motor fluctuation rates between paper and electronic diaries. Spearman correlation coefficient was also used to compare the MDS-UPDRS 1, 2, 3, 4, PDQ-39 and the motor symptoms recorded on paper and electronic diaries, respectively, to examine the validity of the recorded symptoms. To evaluate whether the diary type and number of days were associated with changes in the number of entries recorded, an analysis of covariance (ANCOVA) was performed. The ANCOVA model included the diary type (either paper diary or electronic diary) and the number of days as independent variables, with the number of entries as the dependent variable. Statistical analysis was performed using the R software.^1^ The level of significance was set at *p* < 0.05.

## Results

The number of participants was 19. A total of 17 participants were analyzed, excluding one who entered the data only once during the 7-day paper and digital diary recording period, respectively, and one whose paper symptom diary was illegible. The median age was 61 years (IQR 48–64) and 10 (59%) were male. Detailed basic characteristics are shown in Table 1.

**Table 1:** Baseline characteristics of patients with Parkinson’s disease.

	Patients (*n* = 17)
Age, years (IQR)	61 (48–64)
Sex, *n* (%)	
Male	10 (59)
Female	7 (41)
Duration, year (IQR)	8 (6–10)
Hoehn and Yahr, *n* (%)	
2	13 (77)
3	3 (18)
4	1 (6)
LEDD, mg (IQR)	1,050 (600–1,510)
MMSE, *n* (%)	
27	2 (12)
28	1 (6)
29	3 (18)
30	11 (65)
MDS-UPDRS 1 (IQR)	7 (4–14)
MDS-UPDRS 2 (IQR)	9 (6–14)
MDS-UDPRS3 3 (IQR)	16 (11–18)
MDS-UPDRS 4 (IQR)	4 (0–11)
PDQ-39 (IQR)	32 (15–54)

IQR, interquartile range; LEDD, levodopa equivalent daily dose; MMSE, mini mental state examination; MDS-UPDRS, Movement Disorder Society-Sponsored Revision of the Unified Parkinson’s Disease Rating Scale; PDQ-39, The Parkinson’s disease questionnaire-39.

Table 2 shows the entry status of the paper and electronic symptom diaries. No significant difference in the number of entries was found between paper and electronic symptom diaries (paper 115 [71–147] vs. electronic 109 [93–116], *p* = 0.77). Reporting time was significantly higher in the paper symptom diary, and “Missing time” was significantly higher in the electronic symptom diary.

**Table 2:** Comparison of recording time for each status in paper and electronic diaries.

	Paper-D.	Electronic-D.	*p*
Total entries, (IQR)	115 (71–147)	107 (93–116)	0.77
Total reported time, h (IQR)	69 (61–80)	48 (33–57)	0.001
Troublesome dyskinesia time, h (IQR)	0 (0–1.5)	0 (0–0.5)	0.63
Troublesome dyskinesia rate, % (IQR)	0 (0.0–0.2)	0 (0–0)	0.81
Total ON time, h (IQR)	49.5 (28.5–56)	33 (24.5–50)	0.12
ON time rate, % (IQR)	67 (62–81)	80 (64–98)	0.44
Total OFF time, h (IQR)	16 (8–28)	5 (0.5–9.5)	0.01
OFF time rate, % (IQR)	25 (13–36)	14 (1–27)	0.34
Total duplicate time, h (IQR)	0 (0–0.5)	0.5 (0.5–2.0)	0.14
Total Missing time, h (IQR)	12.5 (4.5–20)	37.5 (33.5–51.5)	< 0.001

IQR, interquartile range.

**Figure 2:**
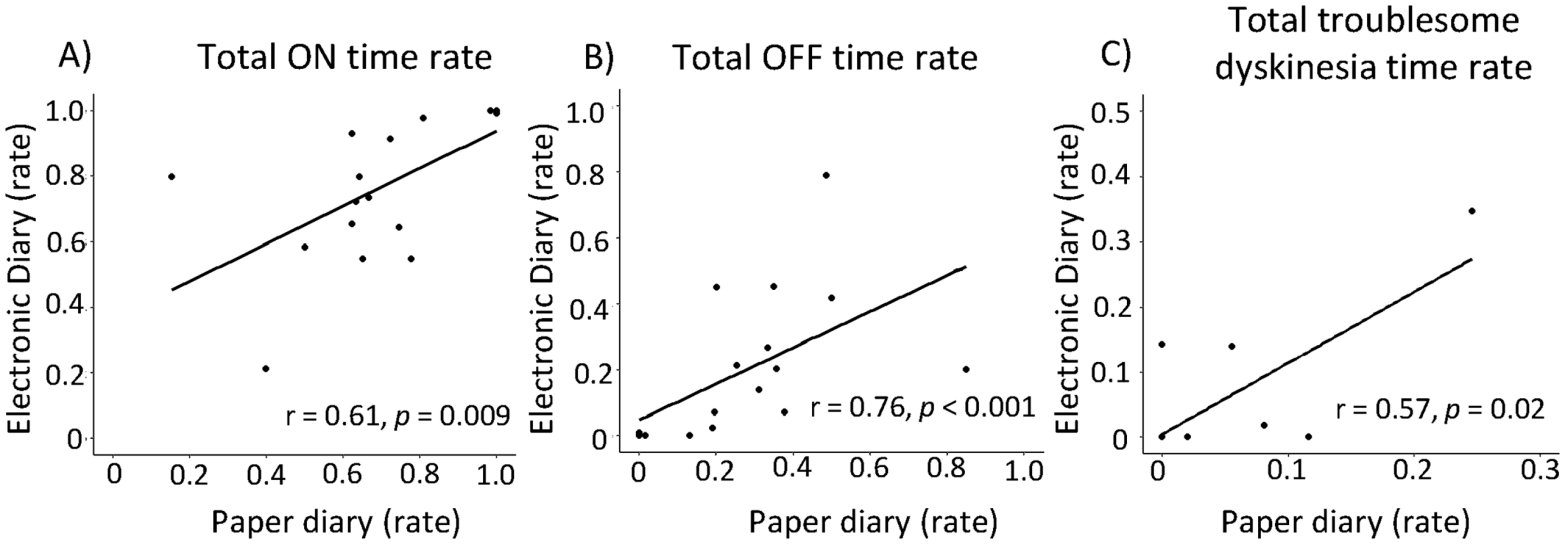
Correlations between electronic diary and paper diary. Correlations between electronic diary and paper diary based on motor status: Total ON time rate (A), total OFF time rate (B), Total troublesome dyskinesia rate (C). There were significant correlations between the proportion of motor status each group.

Next, we compared the motor fluctuation rate (ON-time rate, OFF-time rate, and Troublesome dyskinesia rate) evaluated by paper and electronic diaries (Figure 2). Significant correlations were found between paper and electronic diaries for ON-time rate, OFF-time rate, and troublesome dyskinesia time rate (*r* = 0.61 [*p* < 0.05], *r* = 0.76 [*p* < 0.05], and *r* = 0.57 [*p* < 0 05], respectively). Then, we analyzed the correlation between the status of the motor symptoms captured in each symptom diary and the Parkinson’s disease clinical scales, MDS-UPDRS part 1–4 and PDQ-39 (Table 3). Interestingly, the electronic diary-based ON time rate was significantly correlated with several clinical scales, including MDS-UPDRS part 1, 2, 4, and PDQ-39. The electronic diary-based OFF time rate was also significantly correlated with MDS-UPDRS part 1 and 2. On the other hand, the paper diary-based ON time rate was only significantly correlated with UPDRS part 1 and 4, and the paper diary-based OFF time rate was not significantly correlated with any of the scores.

**Table 3:** Correlations between symptom diaries and scales.

	Electronic-D. ON time-rate	Electronic-D. OFF time-rate	Paper-D. ON time-rate	Paper-D. OFF time-rate
MDS-UPDRS part 1	−0.69**	0.51*	−0.48*	0.38
MDS-UPDRS part 2	−0.58*	0.49*	−0.07	0.10
MDS-UPDRS part 3	0.07	−0.28	0.23	−0.35
MDS-UPDRS part 4	−0.70**	0.47	−0.56*	0.42
PDQ-39	−0.58**	0.40	0.24	0.21

Spearman correlation: **p < 0.01, *p < 0.05.

MDS-UPDRS, Movement Disorder Society-Sponsored Revision of the Unified Parkinson’s Disease Rating Scale; PDQ-39, The Parkinson’s disease questionnaire-39.

**Figure 3:**
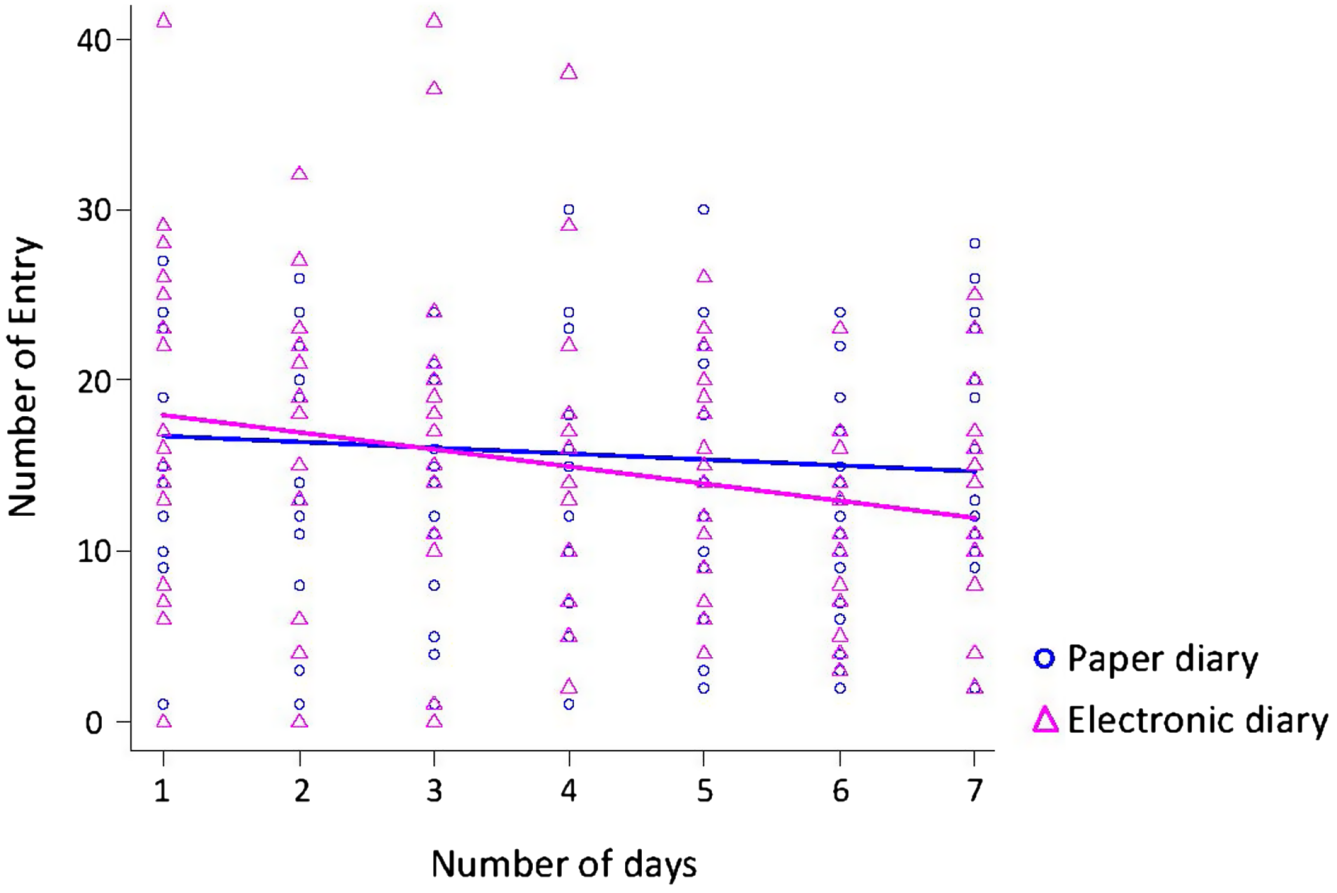
Relationship between the number of entries and the number of days. The number of days was associated with a decrease in the number of entries. Diary type was not significantly associated with a decrease in the number of entries.

Additionally, we analyzed whether the diary type and number of days were associated with changes in the number of entries recorded (Figure 3). The number of days was associated with a decrease in the number of entries (*F*_1,235_ = 6.83, *p* = 0.01). Diary type was not significantly associated with a decrease in the number of entries (*F*_1,235_ = 0.48, *p* = 0.49).

**Figure 4:**
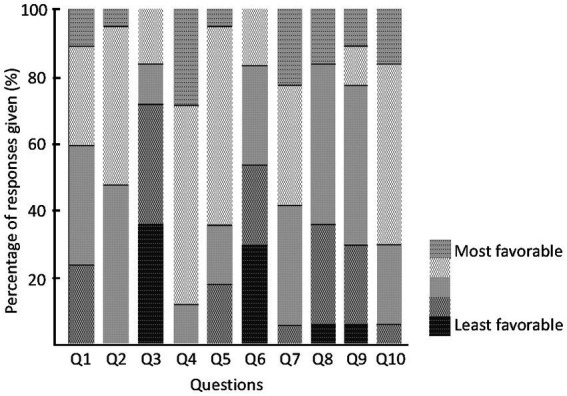
Patients questionnaire. Responses given to the usability questionnaire (%). Color code from green (score = 1 for least favorable response) to orange (score = 5 for most favorable response).

Finally, the results of the patient survey about usability are shown in Figure 4. Regarding daily life interruptions due to the use of the symptom diary, about half of the patients reported that the paper version sometimes or frequently interfered with their daily life (Q1). In contrast, no patient reported that the electronic diary interfered frequently and only 12% of the patients reported that it sometimes interfered with their daily life (Q4). None of the participants found the recording method difficult on paper (Q2). Even with the electronic diary, 80% of patients rated it as operable (Q5).

## Discussion

We developed an electronic diary in Japanese. There was no significant difference in the number of entries between the paper and electronic diaries, maintaining compliance. However, the reported time was significantly shorter for the electronic symptom diaries. This suggests that the paper diary involves recording symptoms retrospectively. Our results showed that about one-fourth of the time recorded in the paper diary was done retrospectively, highlighting the issue of recall bias, which has been identified as a problem with paper symptom diaries. In this study, we showed that the status of the motor symptoms, such as ON time rate, OFF time rate, and Troublesome dyskinesia rate, was significantly correlated between paper and electronic symptom diaries, which is consistent with previous studies. Interestingly, however, when comparing the recorded status of the motor symptoms to other patient-reported outcomes such as MDS-UPDRS part 1, 2, 4 and PDQ-39, the electronic symptom diary showed significant correlations with a wider range of items than the paper symptom diary. PDQ-39 and MDS-UPDRS part 4 have been reported to correlate with motor fluctuations in PD patients (11, 12). MDS-UPDRS part 1 and 2 have been reported to relate to quality of life in PD patients (13). Therefore, the electronic symptom diary may more accurately reflect the patient’s symptoms and quality of life. Our electronic diary, which allowed only real-time entries, may have eliminated recall bias, thereby reflecting the patient’s symptoms more accurately. The reason why MDS-UPDRS par 3 did not correlate with both paper and electronic diary may be due to the fact that MDS-UPDRS part 3 was only evaluated at the time of the outpatient visit, which does not correctly reflect the patient’s motor fluctuation and general status at home. The symptom diary, which records continuous symptoms, is crucial in managing patients with Parkinson’s disease. Our electronic diary offers the advantage of also being able to record and evaluate patient-reported outcomes such as MDS-UPDRS part 1, 2, 4 and PDQ-39.

The usability of our electronic diary was found to be satisfactory based on the results of patients` questionnaire.

The present study also suggests that real-time input is difficult. Missing time in the electronic symptom diary averaged 37.5 h, or 45% of the total time. The devices had reminders to remind them every 30 min with vibration, but patients said that they often did not notice the vibration in their daily life and work, and even when they did notice it, they could not respond immediately, resulting in missed entries. In addition, the electronic symptom diary was sometimes unavailable for a certain period of time due to equipment failure or battery problems with the device. Improvement of the reminding function should be considered in the future. Furthermore, regardless of the type of symptom diary (paper or electronic), the number of entries tended to decrease as the number of days passed, suggesting user fatigue. Despite the reduced recording time, the correlations between exercise symptoms recorded in the electronic diary and patient-reported outcomes such as MDS-UPDRS part 1, 2, 4 and PDQ-39 were strong, suggesting that the recording frequency need not be as frequent as every 30 min. Determining the appropriate recording frequency is a subject for future study.

In addition, this study has several other limitations. The study design included a small sample size and was not a crossover. Conducting the paper diary first, followed by the electronic diary, also introduces potential bias. We acknowledge the need for a larger cross-over study in the future. Another limitation is that the study did not implement the paper and app-based symptom diaries simultaneously, so it was not possible to examine concordance between the two methods for identical epochs. However, since there were no medication changes for Parkinson’s disease during this period and the assessments were conducted within a similar timeframe, we believe that the two methods likely reflect comparable motor fluctuations. Furthermore, the doctor’s evaluation was not conducted simultaneously with the patient reports, so no supervised data were available. In future studies, it would be beneficial to incorporate objective data collection methods, such as accelerometers, to compare against patient-oriented diaries. As previously noted, the correlational analyses were not based on exactly matching time periods, making it difficult to determine whether the reported motor fluctuations are accurate when compared to the patient questionnaire data (MDS-UPDRS and PDQ-39).

In conclusion, our electronic diary is a useful tool that more accurately reflects the patient’s motor symptoms and quality of life compared to the paper symptom diary. In the future, we hope that the use of such digital instruments to assess drug efficacy and DAT responsiveness will enhance more data-driven Parkinson’s disease treatment and ultimately lead to improved patient quality of life.

## Data Availability

The original contributions presented in the study are included in the article/Supplementary material, further inquiries can be directed to the corresponding authors.

## Footnotes

^1^ https://cran.r-project.org/

## Supplementary material

SUPPLEMENTARY FIGURE 1 Examples of each symptom diary. (A) Paper diary. In this case, the diary showed eight entries, and the reported time was 8 h. (B) Electronic dairy. In this case, the diary showed eight entries, and the reported time was 4 h.

SUPPLEMENTARY FIGURE 2 Data protocol. Patients use a smartphone or smartwatch application to record their motor status. The data is transmitted to a server/database and can be shared with doctors through a web application. A reminder function is available on both the smartphone and smartwatch, which sends a push notification if no symptom entries are recorded within a 30-min period.
