# Supplementary figures and images for: Evaluation of motor fluctuations in Parkinson’s disease: electronic vs. conventional paper diaries
Source: Front Neurol. 2024 Nov 22;15:1476708. doi: 10.3389/fneur.2024.1476708 (PMC11622251; doi:10.3389/fneur.2024.1476708)

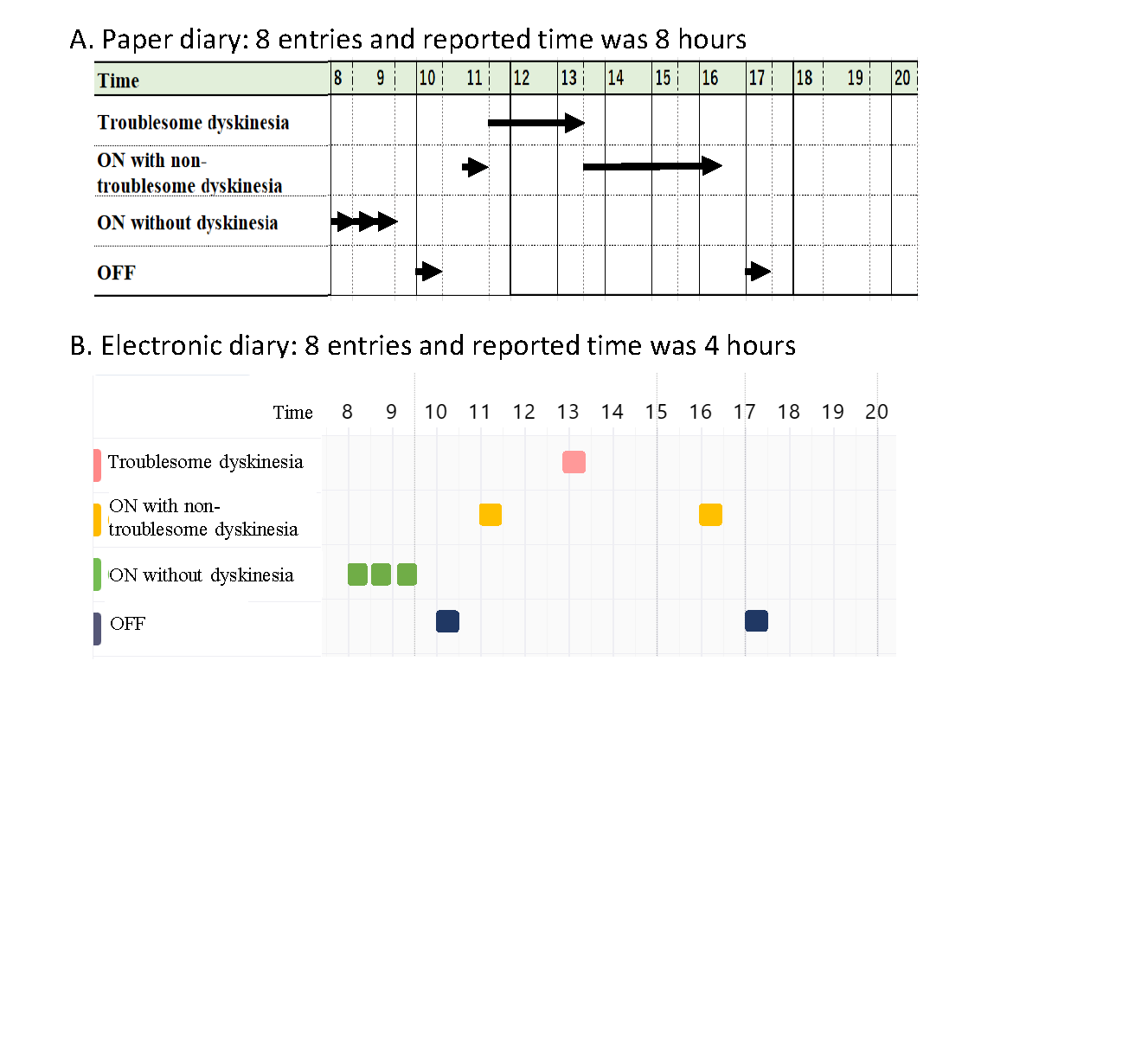

Supplement: SUPPLEMENTARY FIGURE 1 — Examples of each symptom diary. (A) Paper diary. In this case, the diary showed eight entries, and the reported time was 8 h. (B) Electronic dairy. In this case, the diary showed eight entries, and the reported time was 4 h. [file Image_1.TIF]
